# Mettl3 promotes oxLDL‐mediated inflammation through activating STAT1 signaling

**DOI:** 10.1002/jcla.24019

**Published:** 2021-11-26

**Authors:** Zhenwei Li, Qingqing Xu, Ning Huangfu, Xiaomin Chen, Jianhua Zhu

**Affiliations:** ^1^ Department of Cardiology The first Affiliated Hospital Zhejiang University School of Medicine Hangzhou China; ^2^ Department of Cardiology Ningbo Hospital of Zhejiang University Ningbo China; ^3^ Department of Nephrology Ningbo First Hospital Ningbo China

**Keywords:** atherosclerosis, inflammation, methyltransferase‐like protein 3, N6‐methyladenosine, signal transducer and activator of transcription 1

## Abstract

**Background:**

Atherosclerosis (AS) is the main cause of cerebrovascular diseases, and macrophages act important roles during the AS pathological process through regulating inflammation. Modification of the novel N(6)‐methyladenine (m6A) RNA is reported to be associated with AS, but its role in AS is largely unknown. The aim of this study was to investigate the role and mechanism of m6A modification in inflammation triggered by oxidized low‐density lipoprotein (oxLDL) in macrophages during AS.

**Methods:**

RAW264.7 macrophage cells were stimulated with 40 μg/ml ox‐LDL, Dot blot, Immunoprecipitation, western blot, Rip and chip experiments were used in our study.

**Results:**

We found oxLDL stimulation significantly promoted m6A modification level of mRNA in macrophages and knockdown of Methyltransferase‐Like Protein 3 (Mettl3) inhibited oxLDL‐induced m6A modification and inflammatory response. Mettl3 promoted oxLDL‐induced inflammatory response in macrophages through regulating m6A modification of Signal transducer and activator of transcription 1 (STAT1) mRNA, thereby affecting STAT1 expression and activation. Moreover, oxLDL stimulation enhanced the interaction between Mettl3 and STAT1 protein, promoting STAT1 transcriptional regulation of inflammatory factor expression in macrophages eventually.

**Conclusions:**

These results indicate that Mettl3 promotes oxLDL‐triggered inflammation through interacting with STAT1 protein and mRNA in RAW264.7 macrophages, suggesting that Mettl3 may be as a potential target for the clinical treatment of AS.

## Graphical abstract

oxLDL induces LOX‐1 expression and Mettl3 does not affect cell biological activity. (A) qRT‐PCR analysis of LOX‐1 mRNA expression in RAW264.7 cells treated with oxLDL with indicated concentrations for 24 h. (B) WB analysis of LOX‐1 in RAW264.7 cells treated with oxLDL or not for 24 h. (C) WB analysis of the indicated proteins in RAW264.7 cells treated with oxLDL or not for 24 h. (D) Cell viability analysis of RAW264.7 cells transfected with M3 or M3d vector for 24 h. (E) Apoptosis analysis of RAW264.7 cells transfected with M3 or M3d vector for 24 h. (F) Cell viability analysis of RAW264.7 cells transfected with si‐nc or si‐M3 for 24 h. (G) Apoptosis analysis of RAW264.7 cells transfected with si‐nc or si‐M3 for 24 h. (H) Cell cycle analysis of RAW264.7 cells transfected with si‐nc or si‐M3 for 24 h. (I) Cell senescence analysis of RAW264.7 cells transfected with si‐nc or si‐M3 for 24 h.

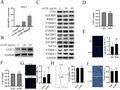

## 1 INTRODUCTION

Atherosclerosis (AS), the major cause of cardiovascular diseases with its main complications, in particular stroke and myocardial infarction, is a chronic inflammatory disease with lipid accumulation and vascular injury.^1^ Owing to more and more aging population and obesity prevalence, the occurrence rate of atherosclerotic disorders is expected to rise over the next decade, and the death number of patient with cardiovascular diseases is expected to reach 23.6 million by 2030.^2^, ^3^ Although significant strides have been made in understanding the underlying mechanism of AS development and treating cardiovascular diseases,^4^, ^5^ safe and effective drugs for preventing and treating AS are still lacking. Thus, deeply and fully elucidating the pathological mechanisms of AS is essential for finding novel drugs for AS.

Nowadays, AS is considered as a chronic inflammatory disease of the arterial wall characterized by lipid accumulation, cell necrosis, and local inflammation.^6^ Macrophages, the major immune cell population in atherosclerotic lesions, play important roles during AS development, from lesion initiation to plaque rupture.^6^, ^7^ In plaque microenvironment, low‐density lipoprotein is not only phagocytized by macrophages to induce the formation of foam cells but also stimulates macrophages to release inflammatory factors such as interleukin‐6 (IL‐6) and tumor necrosis factor alpha (TNF‐α).^8^, ^9^, ^10^ Recent studies revealed that signal transducer and activator of transcription 1 (STAT1), a novel therapeutical target for AS, act crucial roles in the pathology of AS through promoting expressions of several pro‐inflammatory and pro‐atherogenic mediators in macrophages.^11^ Targeted inhibition of STATs and IRFs was reported to be a potential treatment strategy in cardiovascular disease. Thus, understanding macrophage inflammation during AS is a prerequisite to develop novel therapeutic strategies for AS.

N6‐methyladenosine (m6A) is the most abundant internal modification of RNA in eukaryotic cells, and is proved to regulate multiple aspects of RNA metabolism, including RNA processing, nuclear export, RNA translation, and RNA decay.^12^, ^13^ The m6A modification is marked by “writers,” including Methyltransferase‐Like Protein 3 (Mettl3), Mettl14, and the associated protein Wilms' Tumor 1‐Associated Protein (WTAP), and is removed by “erasers,” such as fat mass and obesity‐associated protein (FTO) and AlkB Homolog 5 (ALKBH5).^14^, ^15^, ^16^ The m6A modified RNA is recognized by “readers” to regulate gene expression.^17^, ^18^ Recent study finds that m6A modification levels of RNA are differentially regulated in atherosclerotic lesions.^19^ Besides, Mettl14 is proved to promote endothelial inflammation and AS development,^20^ suggesting that m6A modification is associated with AS development. However, whether m6A modification is involved in inflammation induced by macrophages during AS is still unknown.

In this study, we examined the m6A modification level of mRNA in oxLDL‐stimulated macrophages, and the expressions of m6A “writers.” We found that oxLDL stimulation significantly promoted m6A modification level of mRNA and Mettl3 expression in macrophages. Knockdown of Mettl3 inhibited oxLDL‐induced m6A modification and inflammatory response. Mettl3 enhances oxLDL‐triggered inflammation through promoting STAT1 expression in a m6A‐dependent manner, and Mettl3 interacts with STAT1 to promote STAT1 transcriptional regulation of inflammatory factor expression in RAW264.7 macrophages. In addition, higher m6A modification level and upregulation of Mettl3 protein and corresponding inflammatory factors were detected in monocytes from patients with angiographically proven coronary artery disease (CAD), compared to healthy donors. Overall, our study suggests that Mettl3 may be a potential target for the clinical treatment of AS.

## 2 METHODS

### 2.1 Cell culture and treatment

RAW264.7 macrophage cells were purchased from the American Type Culture Collection (ATCC, USA) and grown in Dulbecco's Modified Eagle's Medium (DMEM) (Sigma, USA) containing 10% fetal bovine serum (FBS) and antibiotics (100 U/ml penicillin A and streptomycin, respectively) at atmosphere of 5% CO_2_ in a 37°C humidified incubator. Cells at 80% confluence were stimulated with 40 μg/ml ox‐LDL (Sigma, USA) for 24 h.

### 2.2 Blood sample collections

In all subjects, blood samples were collected in the morning under fasting state. Human MNCs were isolated from peripheral blood from 16 patients with angiographically proven CAD and 8 healthy donors from Ningbo First Hospital. The study was approved by the research ethics review committee of Ningbo First Hospital, and subjects gave written informed consent before entering the study. Human monocytes were isolated from the peripheral blood by density gradient centrifugation with Histopaque‐1077 (1.077 g/ml, Sigma, USA). The monocytes were cultured in RPMI1640 medium with 10% FBS at atmosphere of 5% CO_2_ in a 37°C humidified incubator.

### 2.3 Total RNA isolation and quantitative real‐time polymerase chain reaction (qRT‐PCR)

After treatment, total RNA of RAW264.7 macrophage cells was extracted using the Trizol reagent (Invitrogen, USA), and then reverse‐transcribed into cDNA using the PrimeScript RT reagent Kit (TaKaRa, Japan). qRT‐PCR was performed using SYBR‐green PCR Master Mix in a Fast Real‐time PCR 7500 System (Applied Biosystems). The sequences of RT‐PCR primer were as following: Mettl3 (forward: 5′‐CTGGGCACTTGGATTTAAGGAA‐3′; reverse: 5′‐TGAGAGGTGGTGTAGCAACTT‐3′); Mettl14 (forward: 5′‐GAGCTGAGAGTGCGGATAGC‐3′; reverse: 5′‐GCAGATGTATCATAGGAAGCCC‐3′); Wtap (forward: 5′‐ATGGCACGGGATGAGTTAATTC‐3′; reverse: 5′‐ATGGCACGGGATGAGTTAATTC‐3′); IL‐6 (forward: 5′‐CTGCAAGAGACTTCCATCCAG‐3′; reverse: 5′‐AGTGGTATAGACAGGTCTGTTGG‐3′); TNF‐α (forward: 5′‐CAGGCGGTGCCTATGTCTC‐3′; reverse: 5′‐CGATCACCCCGAAGTTCAGTAG‐3′); STAT1 (forward: 5′‐TCACAGTGGTTCGAGCTTCAG‐3′; reverse: 5′‐CGAGACATCATAGGCAGCGTG‐3′); oxidized low density lipoprotein (lectin‐like) receptor 1 (LOX‐1) (forward: 5′‐CAAGATGAAGCCTGCGAATGA‐3′; reverse: 5′‐ACCTGGCGTAATTGTGTCCAC‐3′); GAPDH (forward: 5′‐TGGATTTGGACGCATTGGTC‐3′; reverse: 5′‐TTTGCACTGGTACGTGTTGAT‐3′). GAPDH was used as the internal control of the mRNA. Fold changes of target mRNA normalized to a control sample was calculated using the 2^−ΔΔCt^ method.

### 2.4 Western blot

After treatment, total cellular proteins were lysed by RIPA buffer, harvested, and then quantified by bicinchoninic acid (BCA) analysis (Beyotime, China). Protein extractions were separated by 10% SDS‐PAGE and transferred onto polyvinylidene fluoride (PVDF) membranes (Millipore, USA). After the incubation with primary antibodies, including anti‐Mettl3 antibody (1:1000, Abcam, USA), anti‐Mettl14 antibody (1:1000, Abcam, USA), anti‐WTAP antibody (1:1000, Abcam, USA), anti‐flag antibody (1:1000, Abcam, USA), anti‐phosphorylated p38 antibody (p‐p38, 1:1000, Abcam, USA), anti‐p38 antibody (1:1000, Proteintech, China), anti‐phosphorylated JNK antibody (p‐JNK, 1:1000, Abcam, USA), anti‐JNK antibody (1:1000, Proteintech, China), anti‐phosphorylated p65 antibody (p‐p65, 1:1000, Abcam, USA), anti‐p65 antibody (1:1000, Proteintech, China), anti‐phosphorylated STAT1 antibody (p‐STAT1, 1:1000, Abcam, USA), anti‐STAT1 antibody (1:1000, Proteintech, China), anti‐phosphorylated STAT3 antibody (p‐STAT3, 1:1000, Abcam, USA), anti‐STAT3 antibody (1:1000, Proteintech, China), anti‐LOX‐1 antibody (1:1000, Proteintech, China), anti‐FTO antibody (1:1000, Proteintech, China), anti‐ALKBH5 antibody (1:1000, Proteintech, China), anti‐RBM15 antibody (1:1000, Proteintech, China), anti‐YTHDF1 antibody (1:1000, Proteintech, China), anti‐YTHDF2 antibody (1:1000, Proteintech, China), anti‐YTHDF3 antibody (1:1000, Proteintech, China), anti‐IGF2BP1 antibody (1:1000, Proteintech, China), anti‐IGF2BP2 antibody (1:1000, Proteintech, China), anti‐IGF2BP3 antibody (1:1000, Proteintech, China), anti‐EIF3A antibody (1:1000, Proteintech, China), anti‐YTHDC1 antibody (1:1000, Proteintech, China), anti‐YTHDC2 antibody (1:1000, Proteintech, China), and anti‐GAPDH antibody (1:1000, Proteintech, China), the membranes were then incubated with peroxidase (HRP)‐conjugated secondary antibody. After washes with PBS, protein bands were detected using a chemiluminescence system (Bio‐Rad, USA) and analyzed using Image Lab Software.

### 2.5 Cell transfection

Small interfering RNA (siRNA)‐against Mettl3 (si‐M3), siRNA‐against STAT1 (si‐ST1), and control siRNA were synthesized by GenePharma Company (Shanghai, China). A series of truncated mutants of Mettl3, including M3d (which has the mutation of C to S at the position of 314 amino acid, resulting in nearly abolishing the methyltransferase activity^21^), ΔN (which encompasses residues 259 to 580), and ΔNd (which has the mutation of C to S at the position of 314 amino acid in ΔN mutant) were synthesized by GenePharma Company (Shanghai, China), and then cloned into pCMV‐Flag‐vector. siRNAs or plasmids were transfected into Raw264.7 cells with lipofectamine 3000 (Invitrogen) under the guidance of operation instructions. Total RNA and protein were collected 48 h post‐transfection for further experimental analyses.

### 2.6 RNA m6A dot blot assays

After treatment, RNA was extracted with mirVana RNA Isolation Kit (Thermo Scientific) and purified with Dynabeads mRNA Purification Kit (Thermo Scientific). mRNA (40 ng) was denatured by heating at 65°C for 5 min, transferred onto a nitrocellulose membrane with a Bio‐Dot apparatus (Bio‐Rad, USA), UV cross‐linked, blocked with 5% non‐fat milk for 1 h at room temperature, and incubated with anti‐m6A antibody (1:350, Abcam, USA) at 4°C overnight. After washes, the membrane was incubated with HRP‐conjugated secondary antibody, treated with ECL substrate and developed using film.

### 2.7 Enzyme‐linked immunosorbent assay

After treatments, the protein expressions of IL‐6 or TNF‐α in the cell culture supernatants were detected by the Mouse IL‐6 enzyme‐linked immunosorbent assay (ELISA) Kit (Abcam, USA), the Human IL‐6 ELISA Kit (Abcam, USA), the Mouse TNF alpha ELISA Kit (Abcam, USA), or the Human TNF alpha ELISA Kit (Abcam, USA) according to the manufacturer's instruction.

### 2.8 Cell viability assay

After treatments, cell viability of macrophages was detected by the Cell Counting Kit‐8 (Beyotime, China) according to the manufacturer's instruction.

### 2.9 Cell apoptosis assay

Cell apoptosis was measured using the TUNNEL staining kit (Beyotime, China) according to the manufacturer's instructions.

### 2.10 Cell‐cycle analysis

The trypsinized cells (1 × 10^6^) were fixed with 75% ethanol at 4°C for 24 h. The fixed cells were incubated with RNase A (for 30 min at 374°C) after being washed with PBS, and 5 ml of PI (Beyotime, China) was added to the cell suspension. The mixture was incubated at room temperature for 30 min in the dark. The suspended cells were analyzed for cell cycle using flow cytometry (FACSCelesta 2, BD Biosciences). The percentages of cells in the G0/G1, S, and G2/M phases were counted and compared.

### 2.11 Cell senescence analysis

Cell senescence was measured using the Fluorescein di‐beta‐Dgalactopyranoside kit (Biolite, China) by fluorescence microscope according to the manufacturer's instructions.

### 2.12 RNA immunoprecipitation

RNA immunoprecipitation (RIP) assay was performed with Magna RIP Kit (Millipore, USA) following the manufacturer's instructions. In brief, magnetic beads were mixed with 5 μg anti‐m6A antibody (Abcam, USA), anti‐Mettl3 antibody (Abcam, USA), anti‐flag antibody (Abcam, USA), or anti‐mouse/rabbit IgG (Abcam, USA) before the addition of cell lysates (approximately 2 × 10^7^ cells for each sample). After the treatment of proteinase K, interested RNAs were eluted from immunoprecipitated complex and purified for further analysis using qPCR. Immunoprecipitated RNA was analyzed through qRT‐PCR.

### 2.13 Co‐Immunoprecipitation

Co‐Immunoprecipitation (Co‐IP) assay was performed using Dynabeads™ Co‐Immunoprecipitation Kit (ThermoFisher USA), according to the manufacturer's instruction. Briefly, the protein extracted with IP Lysis Buffer was subject to beads premixed with anti‐Mettl3 antibody (Abcam, USA) or IgG. The immunoprecipitated protein complex was separated from beads after several washes, followed by the identification for partners of Mettl3 by immunoblots.

### 2.14 Chromatin immunoprecipitation assay

Chromatin immunoprecipitation (ChIP) assay was performed with Magna ChIP™ A/G kit (Millipore, USA) according to manufacturer's instruction. In brief, 1 × 10^7^ cells fixed with formaldehyde were collected and subject to 500 μl lysis buffer. Then lysate was sonicated, and then the supernatant was diluted and fully mixed with Protein A/G magnetic beads. Then 5 μg of anti‐Mettl3 antibody (Abcam, USA), anti‐STAT1 antibody (Abcam, USA), or IgG was added respectively, followed by incubation at 4°C overnight. The next day, after washing, the mixture was incubated with elution buffer at 62°C for 2 h and then at 95°C for 10 min. Then DNA was purified from the elution and was subject to RT‐qPCR with primers: IL‐6 promoter (forward: 5′‐GTTCCTGGTTTTCTGTCCACCT‐3′; reverse: 5′‐ATGTACACTAAGTCCACCCATG‐3′). Relative enrichment was normalized to the input: %Input = 1/10 × 2^Ct [IP] – Ct [input]^.

### 2.15 Statistical analysis

Data were analyzed using SPSS version 19.0 and presented as means ± standard deviation (SD). Student's *t*‐test was performed to analyze differences between the two groups and ANOVA (parametric) was performed to analyze differences among multiply groups. *p* < 0.05 was considered to indicate statistical significance.

## 3 RESULTS

### 3.1 oxLDL stimulation significantly increases m6A modification levels in macrophages and knockdown of Mettl3 inhibits oxLDL‐induced m6A modification and inflammatory response

**FIGURE 1:**
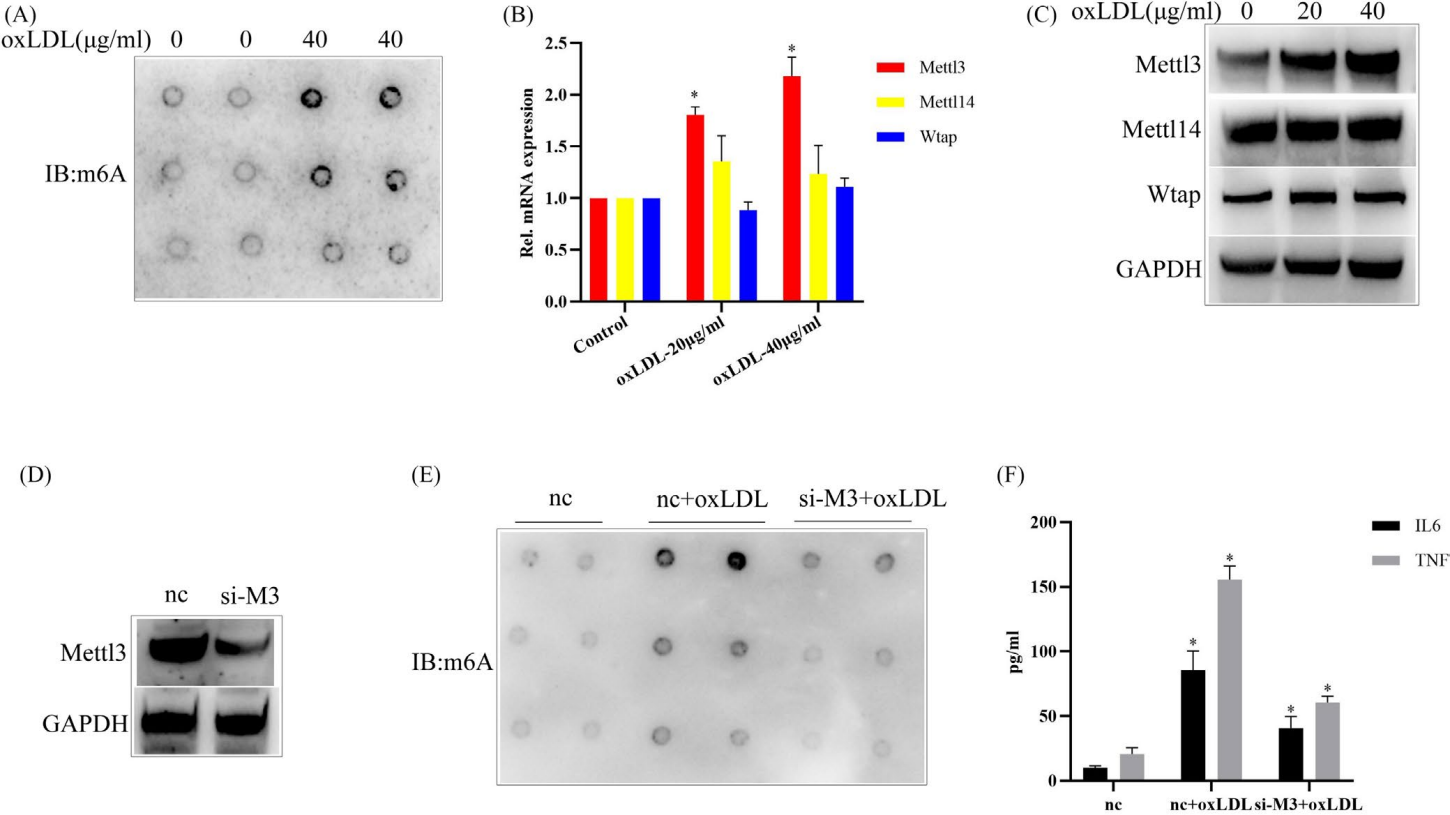
oxLDL stimulation significantly promotes m6A modification level of RNA in macrophages and knockdown of mettl3 inhibits oxLDL‐induced m6A modification and inflammatory response. (A) Dot blot quantification of m6A abundance in the mRNA isolated from the RAW264.7 macrophage cells treated with 40 μg/ml oxLDL or not for 24 h. (B) qRT‐PCR analysis of the indicated genes in RAW264.7 cells treated with oxLDL or not for 24 h. (C) WB analysis of the indicated proteins in RAW264.7 cells treated with oxLDL or not for 24 h. (D) WB analysis of the effect of siRNA‐Mettl3 (si‐M3). NC is the negative control. (E) Dot blot quantification of m6A abundance in the mRNA isolated from the RAW264.7 macrophage cells transfected with si‐M3 and treated with oxLDL. (F) Elisa analysis of the protein expression levels of IL‐6 or TNF‐α in the cell culture supernatants from the RAW264.7 macrophage cells transfected with si‐M3 and treated with oxLDL. Data are represented as means ± SD (*n* = 3; *represents *p* < 0.05)

Recent study reports that aberrant m6A level exists in different stages of atherosclerotic lesions.^19^ To explore whether m6A modification is involved in oxLDL‐induced inflammatory response in macrophages, we first examined the global m6A levels in oxLDL‐stimulated macrophages. As shown in Figure 1A, oxLDL stimulation significantly promotes m6A modification level of RNA in macrophages. We further examined the expression levels of m6A “writers,” and found that oxLDL stimulation evidently increased the mRNA and protein expression levels of Mettl3 and LOX‐1. However, the expression of Mettl14, WTAP or other m6A modification‐related proteins was not affected (Figure 1B,C, and Figure S1A–C). Moreover, we examined the effects of Mettl3 on the global m6A levels and inflammatory response in oxLDL‐stimulated macrophages. The results indicated that Mettl3 knockdown significantly suppressed oxLDL‐induced upregulation of global m6A levels (Figure 1D,E) and inhibited oxLDL‐induced expressions of pro‐inflammatory factors such as IL‐6 and TNF‐α (Figure 1F). Collectively, the results suggest that oxLDL stimulation significantly promotes m6A modification level of RNA in macrophages and knockdown of Mettl3 inhibits oxLDL‐induced m6A modification and inflammatory response.

### 3.2 Mettl3 promoting oxLDL‐induced inflammatory response in macrophages depends on its methyltransferase activity

**FIGURE 2:**
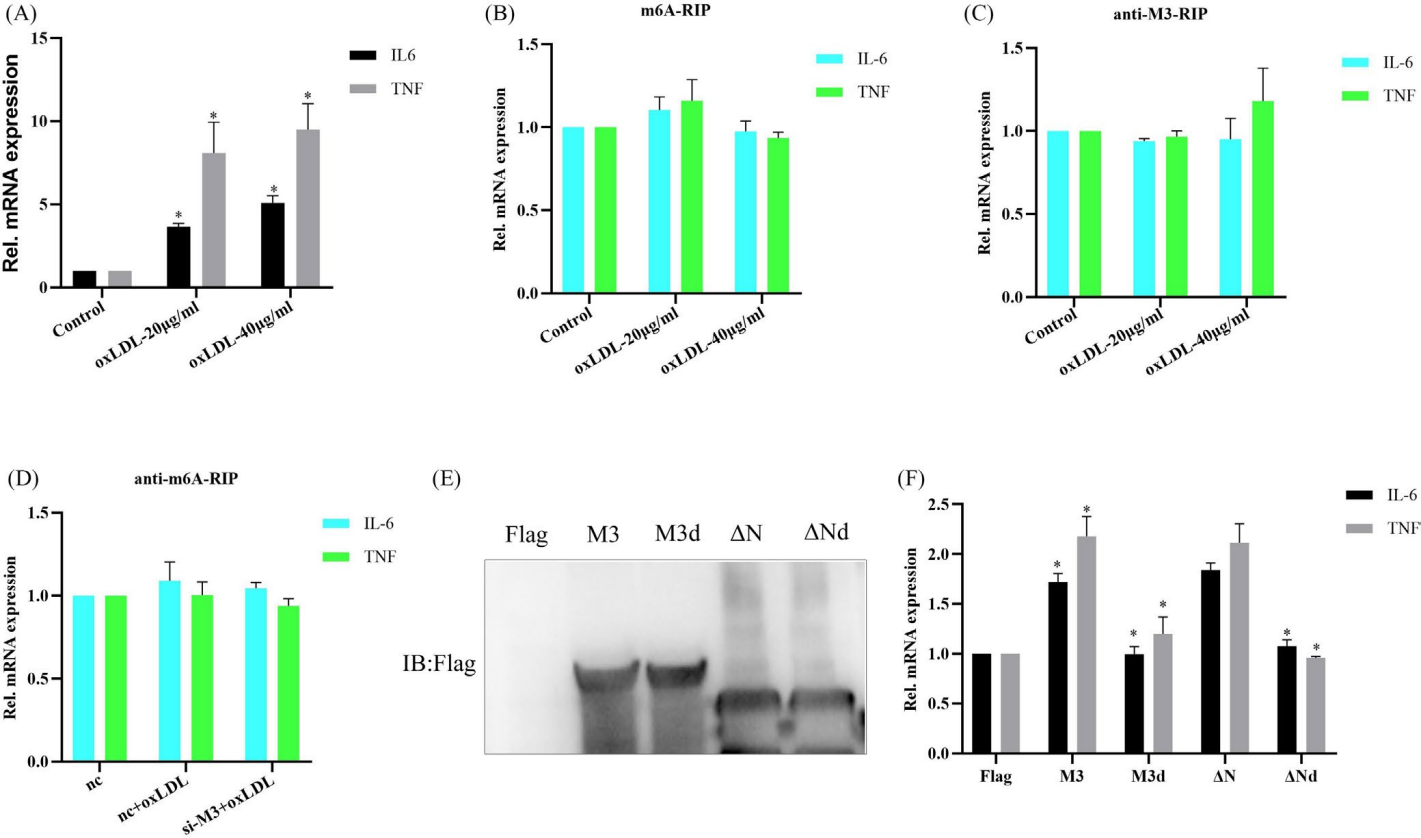
Mettl3 promoting oxLDL‐induced inflammatory response in macrophages depends on its methyltransferase activity. (A) qRT‐PCR analysis of IL‐6 or TNF‐α mRNA expression in RAW264.7 cells treated with different density oxLDL (B) RIP analysis of the m6A modification level on IL‐6 or TNF‐α mRNA in RAW264.7 cells treated with oxLDL or not for 24 h. (C) RIP analysis of Mettl3 (M3) interacting with IL‐6 or TNF‐α mRNA in RAW264.7 cells treated with oxLDL or not for 24 h. (D) RIP analysis of the m6A modification level on IL‐6 or TNF‐α mRNA in RAW264.7 macrophage cells transfected with si‐M3 and treated with oxLDL, (E) WB analysis of protein expressions in RAW264.7 cells transfected with different truncated mutants of flag‐labeled Mettl3. M3, wild type Mettl3 containing 1 to 580 amino acid; M3d has the mutation of C to S at the position of 314 amino acid, resulting in nearly abolishing the methyltransferase activity; ΔN encompasses residues 259 to 580; ΔNd has the mutation of C to S at the position of 314 amino acid in ΔN mutant. (F) qRT‐PCR analysis of IL‐6 or TNF‐α mRNA expression in RAW264.7 cells transfected with different truncated mutants of flag‐labeled Mettl3. Data are represented as means ± SD (*n* = 3; *represents *p* < 0.05)

M6A modification of mRNA is related to regulation of gene expression.^21^ qRT‐PCR analysis showed that oxLDL treatment significantly increased IL‐6 or TNF‐α mRNA expression in macrophages (Figure 2A). To further explore the underlying mechanism of Mettl3 promoting oxLDL‐induced pro‐inflammatory factor expression in macrophages, we examined whether Mettl3 affected m6A modification on pro‐inflammatory factor, IL‐6, and TNF‐α mRNA by RIP assay. The results showed that oxLDL stimulation did not affect m6A modification or Mettl3 binding to IL‐6/TNF‐α mRNA (Figure 2B,C). Furthermore, Mettl3 knockdown did not affect m6A modification on IL‐6/TNF‐α mRNA (Figure 2D). Besides, Mettl3 did not affect cell viability, cell apoptosis, cell cycle, or cell senescence (Figure S1F–I). In consideration of that the methyltransferase activity of Mettl3 is essential for regulating gene expression,^20^ we further examined whether the methyltransferase activity of Mettl3 affected IL‐6/TNF‐α mRNA. Next, we constructed a series of truncated mutants of Mettl3, including M3d (which has the mutation of C to S at the position of 314 amino acid, resulting in nearly abolishing the methyltransferase activity^21^), ΔN (which encompasses residues 259 to 580), and ΔNd (which has the mutation of C to S at the position of 314 amino acid in ΔN mutant) (Figure 2E). qRT‐PCR analysis showed that overexpression of Mettl3 or Mettl3 ΔN mutant significantly promoted IL‐6/TNF‐α mRNA expression, whereas overexpression of Mettl3 M3d or Mettl3 ΔNd mutant did not obviously affect IL‐6/TNF‐α mRNA expression (Figure 2F). In addition, overexpression of Mettl3 or Mettl3 M3d did not affect cell viability or apoptosis (Figure S1D,E). Collectively, the results suggest that Mettl3 can promote oxLDL‐induced inflammatory response in macrophages depends on its methyltransferase activity.

### 3.3 Mettl3 promotes oxLDL‐induced inflammatory response in macrophages through regulating STAT1

**FIGURE 3:**
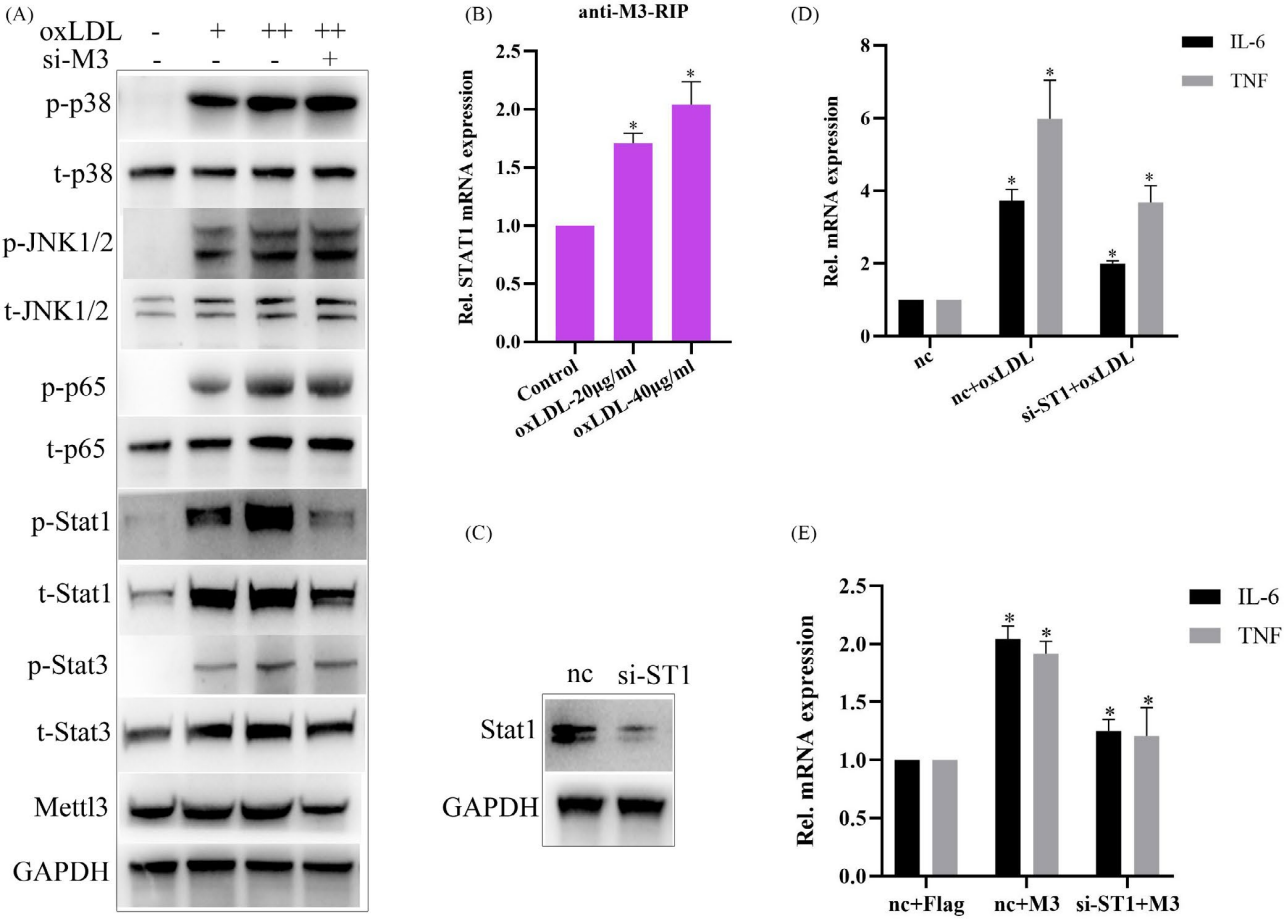
Mettl3 promotes oxLDL‐induced inflammatory response in macrophages through regulating STAT1. (A) WB analysis of the indicated protein expressions in RAW264.7 cells transfected with transfected with si‐M3 and treated with oxLDL. (B) RIP analysis of Mettl3 (M3) interacting with STAT1 mRNA in RAW264.7 cells treated with oxLDL or not. (C) WB analysis of the effect of siRNA‐STAT1 (si‐ST1). NC is the negative control. (D) qRT‐PCR analysis of IL‐6 or TNF‐α mRNA expression in RAW264.7 cells transfected with si‐ST1 and treated with oxLDL. (E) qRT‐PCR analysis of IL‐6 or TNF‐α mRNA expression in RAW264.7 cells transfected with si‐ST1 and pCMV‐flag‐Mettl3 (M3). Data are represented as means ± SD (*n* = 3; *represents *p* < 0.05)

Considering that inflammatory response is usually triggered by mitogen‐activated protein kinase (MAPK) signaling, such as p38 and Jun‐NH(2)‐terminal kinase (JNK), p65 nuclear factor kappa‐light‐chain‐enhancer of activated B cells (NF‐κB) signaling, and Janus kinase (JAK)‐signal transducer and activator of transcription 1/3(STAT1/3) signaling,^22^, ^23^, ^24^, ^25^, ^26^ we further explored whether Mettl3 promoting oxLDL‐induced inflammatory response in macrophages is associated with these signaling pathways. As shown in Figure 3A, oxLDL stimulation significantly increased the protein expressions of phosphorylated p38 (p‐p38), p‐JNK1/2, p‐p65, p‐STAT1, and p‐STAT3, while Mettl3 knockdown obviously suppressed the upregulation of total STAT1 and p‐STAT1 protein expressions. Furthermore, we found that oxLDL stimulation significantly enhanced m6A modification of STAT1 mRNA (Figure 3C). Moreover, knockdown of STAT1 not only obviously inhibited oxLDL‐induced IL‐6/TNF‐α mRNA expression in macrophages but also suppressed Mettl3 overexpression‐induced IL‐6/TNF‐α mRNA expression (Figure 3D,E). Overall, the results indicate that Mettl3 promotes oxLDL‐induced inflammatory response in macrophages through regulating STAT1.

### 3.4 oxLDL stimulation significantly promoted m6A modification of STAT1 mRNA, and enhanced Mettl3 interacting with STAT1 mRNA

**FIGURE 4:**
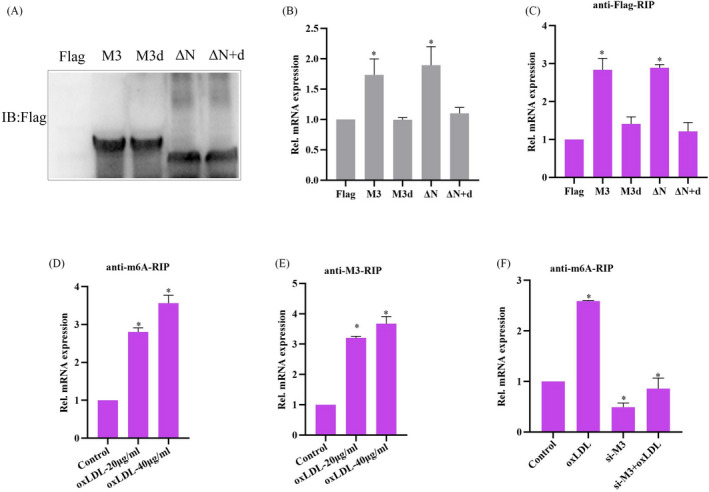
oxLDL stimulation significantly promoted m6A modification of STAT1 mRNA, and enhanced Mettl3 interacting with STAT1 mRNA. (A) WB analysis of protein expressions in RAW264.7 cells transfected with different truncated mutants of flag‐labeled Mettl3 (Left). qRT‐PCR analysis of STAT1 mRNA expression in RAW264.7 cells transfected with different truncated mutants of flag‐labeled Mettl3 (Right). (B) RIP analysis of different truncated mutants of flag‐labeled Mettl3 interacting with STAT1 mRNA in RAW264.7 cells. (C) RIP analysis of the m6A modification level on STAT1 mRNA in RAW264.7 cells treated with oxLDL or not for 24 h. (D) RIP analysis of Mettl3 (M3) interacting with STAT1 mRNA in RAW264.7 cells treated with oxLDL or not. (E) RIP analysis of the m6A modification level on STAT1 mRNA in RAW264.7 cells transfected with si‐ST1 and treated with oxLDL. Data are represented as means ± SD (*n* = 3; *represents *p* < 0.05)

Considering that knockdown of Mettl3 inhibit oxLDL‐induced activation of STAT1 (Figure 3A) and Mettl3 has recently been reported to methylate STAT1 mRNA,^27^ we further explore whether methyltransferase activity of Mettl3 is associated with its effect on STAT1 activation. As shown in Figure 4A,B, overexpression of Mettl3 or Mettl3 ΔN mutant interacted with STAT1 mRNA and significantly promoted STAT1 mRNA expression, whereas overexpression of Mettl3 M3d or Mettl3 ΔNd mutant did not show similar effects, suggesting that the methyltransferase activity of Mettl3 is essential for its effects on STAT1 expression. Furthermore, we found that oxLDL stimulation significantly promoted m6A modification of STAT1 mRNA and enhanced Mettl3 interacting with STAT1 mRNA (Figure 4C,D). Knockdown of Mettl3 significantly inhibited m6A modification level of STAT1 mRNA under normal or oxLDL‐stimulated condition (Figure 4E). Overall, the results indicate that Mettl3 promotes STAT1 mRNA expression through regulating m6A modification of STAT1 mRNA.

### 3.5 Mettl3 interacts with STAT1 to promote STAT1 transcriptional regulation of inflammatory factor expression

**FIGURE 5:**
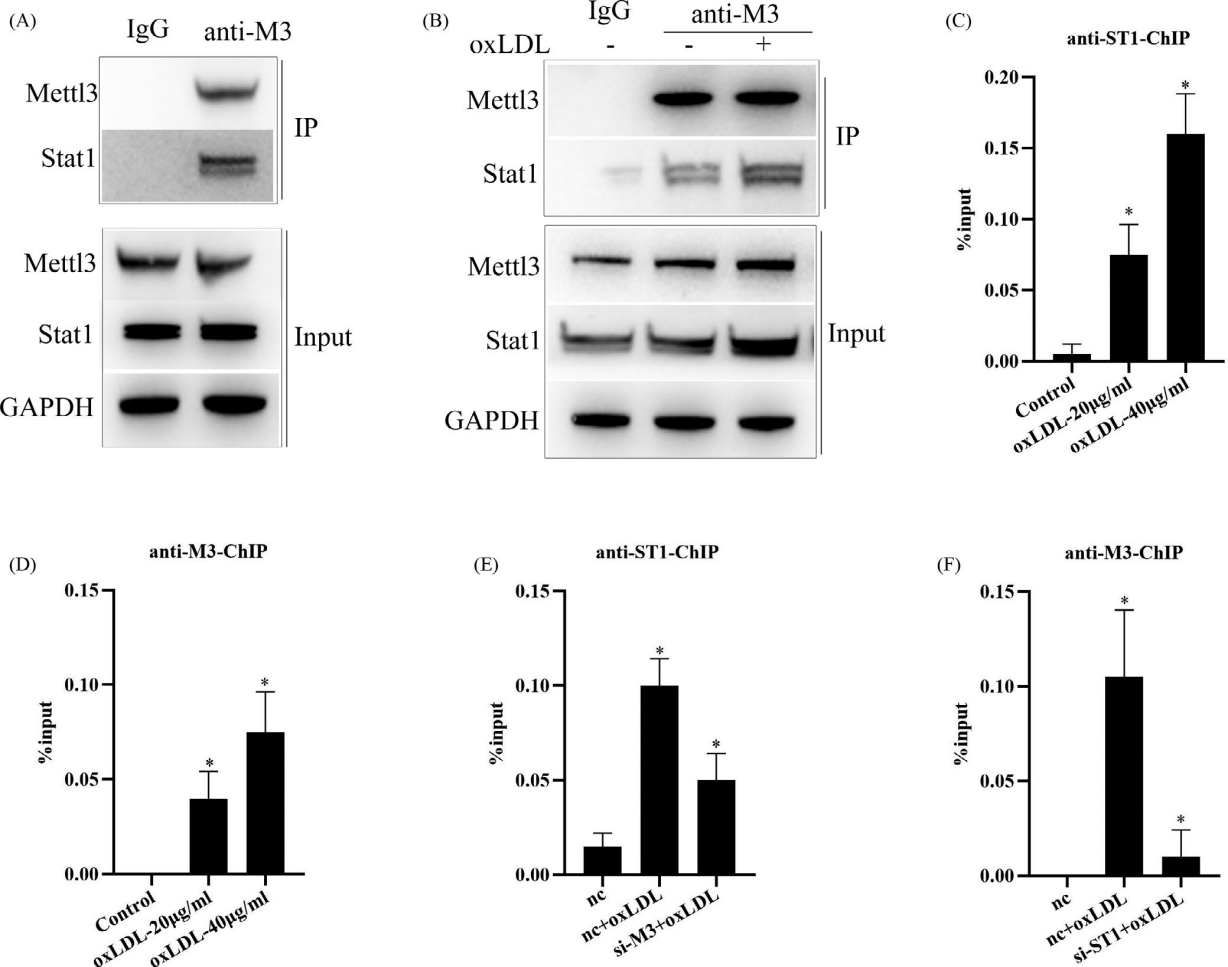
Mettl3 interacts with STAT1 to promote STAT1 transcriptional regulation of inflammatory factor expression. (A) Co‐IP analysis of Mettl3 interacting with STAT1 in RAW264.7 cells. (B) Co‐IP analysis of Mettl3 interacting with STAT1 in RAW264.7 cells treated with oxLDL or not. (C) Chip analysis of STAT1 interacting with IL‐6 gene promoter region in RAW264.7 cells treated with oxLDL. (D) Chip analysis of Mettl3 interacting with IL‐6 gene promoter region in RAW264.7 cells treated with oxLDL. (E) Chip analysis of STAT1 interacting with IL‐6 gene promoter region in RAW264.7 cells transfected with si‐M3 and treated with oxLDL. (F) Chip analysis of Mettl3 interacting with IL‐6 gene promoter region in RAW264.7 cells transfected with si‐ST1 and treated with oxLDL. Data are represented as means ± SD (*n* = 3; *represents *p* < 0.05)

STAT1 is a well‐known master transcription factor controlling expressions of inflammatory factors, such as IL‐6 and TNF‐α.^28^, ^29^ We checked whether Mettl3 could also affect the transcriptional binding of STAT1. The results of Co‐IP showed that Mettl3 interacted with STAT1 in macrophages under normal condition and oxLDL stimulation further increased the interaction of Mettl3 and STAT1 (Figure 5A,B). Furthermore, we found that oxLDL stimulation significantly promoted STAT1 and Mettl3 binding to IL‐6 gene promoter through ChIP analysis (Figure 5C,D). Moreover, knockdown of Mettl3 obviously decreased STAT1 binding to IL‐6 gene promoter (Figure 5E) and knockdown of STAT1 also significantly decreased Mettl3 binding to IL‐6 gene promoter (Figure 5F), suggesting that the Mettl3‐ STAT1 complex is essential for transcriptional regulation of STAT1. Overall, the results indicate that Mettl3 interacts with STAT1 to promote STAT1 transcriptional regulation of inflammatory factor expression.

### 3.6 Mettl3 interacts with STAT1 to promote inflammatory factor expression in monocytes from patients with angiographically proven CAD

**FIGURE 6:**
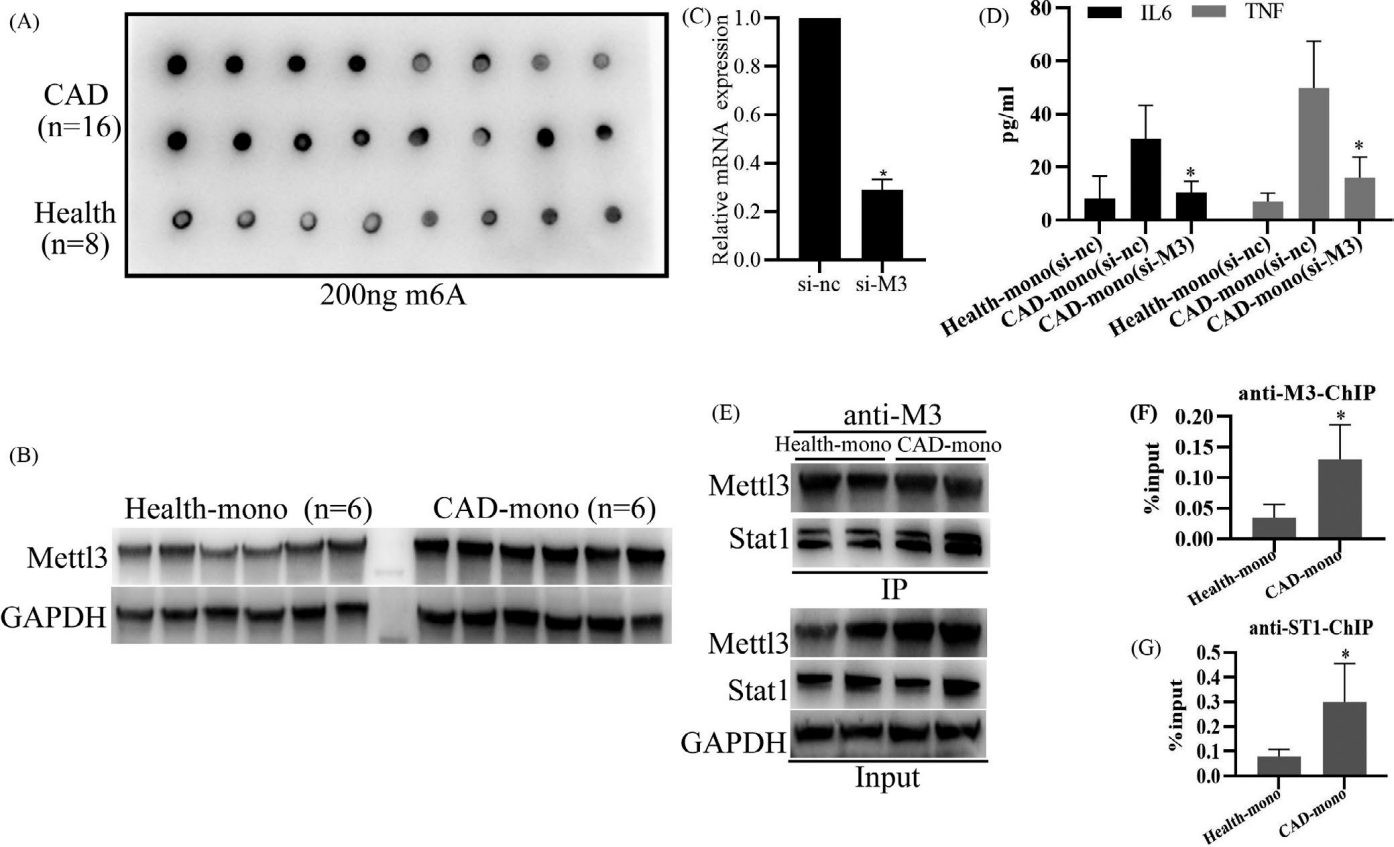
Mettl3 interacts with STAT1 to promote inflammatory factor expression in monocytes from patients with angiographically proven coronary artery disease (CAD). (A) Dot blot quantification of m6A abundance in the mRNA isolated from the monocytes from patients with CAD (*n* = 16) or healthy donors (*n* = 8). (B)WB analysis of Mettl3 protein expression in monocytes from patients with CAD (*n* = 6) or healthy donors (*n* = 6). (C) qRT‐PCR analysis of Mettl3 genes in PBMCs transfected with si‐nc or si‐Mettl3 (si‐M3) for 24 h. (D) Elisa analysis of the protein expression levels of IL‐6 or TNF‐α in the cell culture supernatants of monocytes from patients with CAD or healthy donors transfected with si‐M3. (E) Co‐IP analysis of Mettl3 interacting with STAT1 in monocytes from patients with CAD or healthy donors. (F) Chip analysis of Mettl3 interacting with IL‐6 gene promoter region in monocytes from patients with CAD or healthy donors. (G) Chip analysis of STAT1 interacting with IL‐6 gene promoter region in monocytes from patients with CAD or healthy donors. Data are represented as means ± SD (*n* = 3; *represents *p* < 0.05)

To further explore the relationship between m6A modification and inflammatory response in AS, we collected blood samples of patients with angiographically proven CAD and healthy donors in clinical, and then detected the m6A modification changes in monocytes. As shown in Figure 6A,B, m6A modification levels of RNA in monocytes from patients with CAD were significantly higher than those in healthy donors, and Mettl3 protein levels were markedly higher than those in healthy donors, which are consistent with our previous *in‐vitro* results (Figure 1A,C). Furthermore, we found that expressions of pro‐inflammatory factors, IL‐6 and TNF‐α from monocytes of patients with CAD were also significantly higher than those from healthy donors, and knockdown of Mettl3 (Figure 6C) evidently inhibited IL‐6 and TNF‐α expressions (Figure 6D). Moreover, Co‐IP analysis showed that Mettl3 could bind more STAT1 protein in monocytes from patients with CAD than that in healthy donors (Figure 6E). In addition, Chip analysis showed that more Mettl3 and STAT1 protein bound to IL‐6 gene promoter in monocytes from patients with CAD, compared to those in healthy donors (Figure 6F,G). Overall, these results indicate Mettl3 interacts with STAT1 to promote inflammatory factor expression in monocytes from angiographically proven CAD patients.

## 4 DISCUSSION

Atherosclerosis is now generally considered as a chronic inflammatory disorder with the interaction between inflammation and lipids as a major hallmark.^6^ Macrophages, as the most abundant immune cell type in atherosclerotic lesions, play essential roles during all stages of the disease.^7^ Recent study reports m6A modification levels of RNA are differentially regulated in atherosclerotic lesions.^18^ In this study, our group explored the role of Mettl3 in oxLDL‐induced inflammation in macrophages and detected the m6A modification level in monocytes from patients with CAD. By performing RIP and Co‐IP experiments, we demonstrate that Mettl3 promotes oxLDL‐induced IL‐6 and TNF‐α transcription by upregulating STAT1 protein expression and interacting with STAT1 to activate target genes transcription. Our study clarifies the methyltransferase activity and the non‐methyltransferase activity of Mettl3 are involved in STAT3 regulated transcriptional activation during oxLDL‐induced inflammatory responses. As is known, m6A modifications on mRNA were induced by Mettl3‐Mettl4‐Wtap complex,^30^ but in our study only Mettl3 was obviously upregulated by oxLDL treatment. Although the expression Mettl4 and Wtap were not significantly changed under oxLDL stimulation in macrophages, they should also participate in Mettl3‐mediated m6A modifications on STAT1 mRNA. Why only Mettl3, but not other complex components, was regulated by oxLDL? We thought this phenomenon maybe attributes to that Mettl3 also participates in oxLDL‐induced biological activities, which is independent on its methyltransferase activity. Mettl3‐mediated m6A modifications on STAT1 mRNA promote STAT1 protein expression in macrophages. Whether this effect depends on “m6A readers” such as YTHDF1/2/3 ^31^, ^32^, ^33^ or IGF2BP1/2/3 ^34^, ^35^, ^36^ is also unknown. In this study, we find that Mettl3 cooperates with STAT1 to promote its transcriptional activity. Since m6A modifications on nascent mRNA mediated by Mettl3 could form DNA/RNA hybrid with local single‐strand DNA, and DNA/RNA hybrid generally hinders gene transcription ^37^. We hypothesized how Mettl3 coordinates with DNA/RNA hybrid formation and interacting with STAT1, whether STAT1 interacting with Mettl3 affects its methyltransferase activity. All these details need further investigation. Overall, we explored the role of Mettl3 during oxLDL‐induced inflammation in macrophages and in monocytes from patients with CAD, and found the Mettl3‐STAT1 axis was essential for inflammatory factors transcription.

## 5 CONCLUSION

oxLDL stimulation significantly promoted m6A modification level of mRNA and Mettl3 expression in macrophages. Knockdown of Mettl3 inhibited oxLDL‐induced m6A modification and inflammatory response. Mettl3 enhances oxLDL‐triggered inflammation through promoting STAT1 expression in m6A‐dependent manner, and Mettl3 interacts with STAT1 to promote STAT1 transcriptional regulation of inflammatory factor expression in RAW264.7 macrophages, which was also demonstrated in the monocytes from patients with angiographically proven CAD, suggesting that Mettl3 may be a potential target for the clinical treatment of AS.

## Supporting information

Fig S1

## Data Availability

The data used to support the findings of this study are available from the corresponding author upon request.
