# Supplementary figures and images for: Mettl3 promotes oxLDL‐mediated inflammation through activating STAT1 signaling
Source: J Clin Lab Anal. 2021 Nov 26;36(1):e24019. doi: 10.1002/jcla.24019 (PMC8761454; doi:10.1002/jcla.24019)

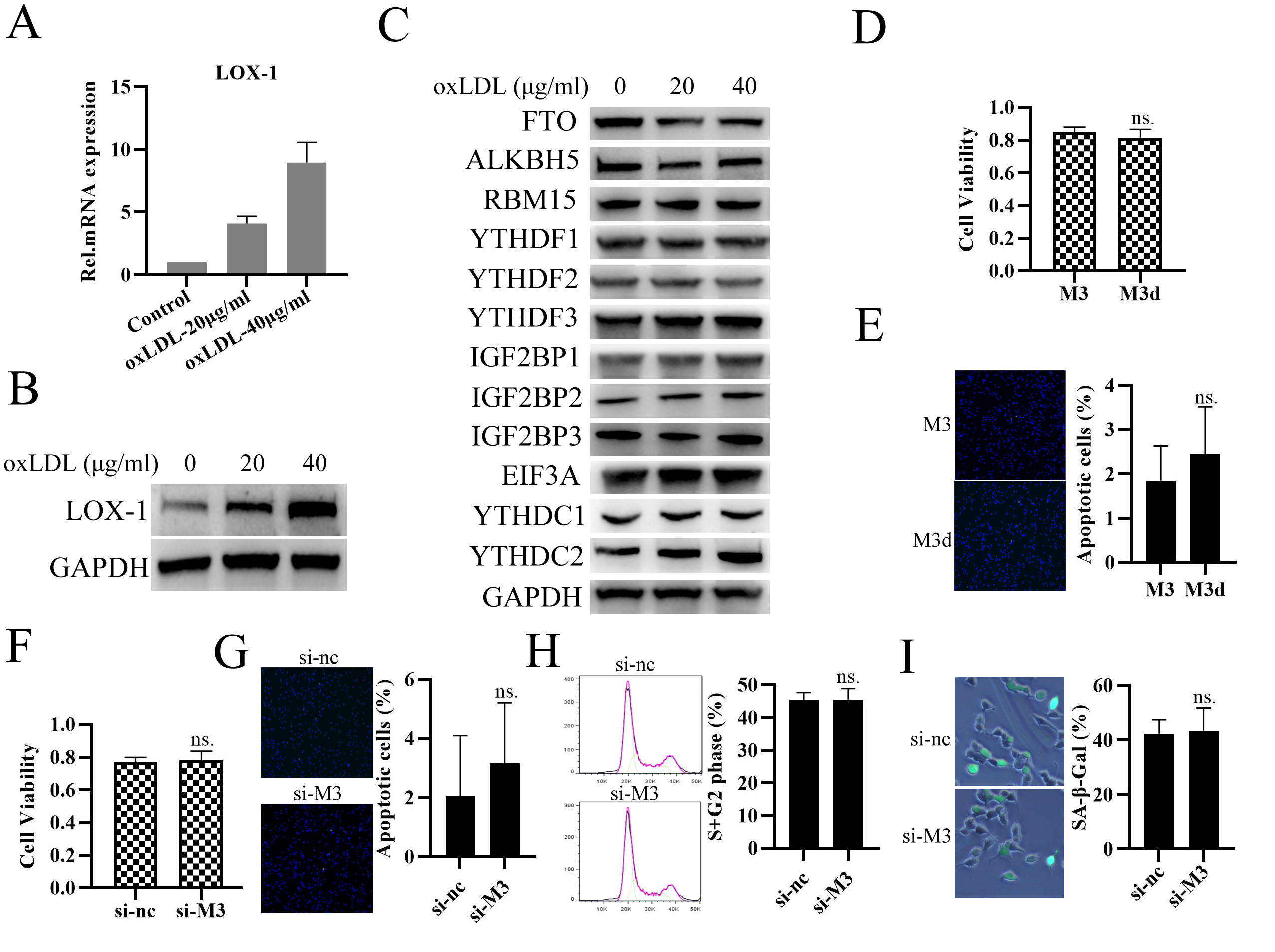

Supplement: Supplementary file 1 — Fig S1 [file JCLA-36-e24019-s001.tif]
